# The brain in chronic insomnia and anxiety disorder: a combined structural and functional fMRI study

**DOI:** 10.3389/fpsyt.2024.1364713

**Published:** 2024-06-04

**Authors:** Minghe Xu, Bo Li, Shuang Wang, Chunlian Chen, Zhe Liu, Yuqing Ji, Kai Liu, Yujun Niu

**Affiliations:** ^1^ Jinzhou Medical University, Jinzhou, China; ^2^ Department of Radiology, The 960th Hospital of People’s Liberation Army Joint Logistic Support Force, Jinan, China

**Keywords:** insomnia disorder, anxiety disorder, comorbidity, brain structure, brain function, magnetic resonance imaging

## Abstract

**Background:**

Chronic insomnia disorder (CID) is usually associated with Generalized Anxiety Disorder (GAD), which may change brain structure and function. However, the possible brain markers, imaging characteristics, and pathophysiology are unknown.

**Objective:**

To look at the probable brain markers, imaging characteristics, and pathogenesis of CID in combination with GAD.

**Methods:**

A total of 57 patients with CID concomitant GAD and 57 healthy controls (HC) were enrolled. Voxel-based morphometry (VBM) and functional connectivity (FC) were utilized to measure gray matter volume (GMV) and functional changes. Correlation analysis was utilized to identify relationships between brain changes and clinical characteristics.

**Results:**

Patients had decreased GMV in the left cerebellum, right cerebellar peduncle, and left insula; increased FC between the left cerebellum and right angular gyrus, as well as between the left insula and anterior left cingulate gyrus; and decreased FC in several areas, including the left cerebellum with the middle left cingulate gyrus and the left insula with the left superior postcentral gyrus. These brain changes related to CID and GAD. These data could be used to identify relevant brain markers, imaging features, and to better understand the etiology.

**Conclusion:**

The intensity of insomnia in patients was strongly related to the severity of anxiety. The lower GMV in the cerebellum could be interpreted as an imaging characteristic of CID. Reduced GMV in the insula, as well as aberrant function in the cingulate gyrus and prefrontal lobe, may contribute to the pathophysiology of CID and GAD. Abnormal function in the postcentral gyrus and angular gyrus may be associated with patients’ clinical complaints.

## 1 Introduction

Insomnia is widely regarded as the most common sleep disorder encountered in clinical practice (1). According to the International Classification of Sleep Disorders, Third Edition, insomnia that persists for more than three months is classified as CID. The primary manifestations of CID include prolonged reduction in sleep duration and diminished sleep quality. GAD is a prevalent clinical mental disorder with a high occurrence rate. It typically arises from excessive anxiety and worry about various events or activities. The main symptoms of GAD include nervousness, heightened alertness, and sleep disturbances. Epidemiological studies suggest that most individuals will experience increased sleep-related anxiety following chronic insomnia (2), often comorbid with GAD (3). The Diagnostic and Statistical Manual of Mental Disorders, Fifth Edition, also recognizes insomnia as a significant clinical indicator and a diagnostic criterion for GAD, with most patients suffering from both CID and GAD. This comorbidity is frequently observed in epidemiological research and may stem from a shared pathogenesis (4). For instance, individuals with both disorders may exhibit abnormal levels of neurotransmitters such as 5-hydroxytryptamine (5, 6), γ-aminobutyric acid (7) and dopamine (8), which maintain a state of hyperarousal and stress (9). Moreover, elevated cortisol levels and dysfunction in the hypothalamic-pituitary-adrenal axis (10) may predispose these patients to a heightened stress response and disrupted sleep (11). Abnormalities in brain regions responsible for sleep and emotional regulation further contribute to both CID and GAD. However, the precise mechanisms underlying this comorbidity remain unclear and are currently under investigation. In this study, we innovatively focus on patients with comorbid insomnia and anxiety, a group commonly represented in empirical research (12). Previous studies have documented this comorbidity extensively: Many patients seeking treatment for insomnia report concurrent anxiety disorders (13), and similarly, individuals with anxiety frequently experience insomnia (14), as confirmed by our correlation analysis.

Voxel-based morphometry (VBM) analysis is widely utilized in brain research to measure the volume and density of brain regions. We selected this method is to identify neural markers of brain structure, building on prior research. Patients with CID and GAD often show brain structural abnormalities resulting from prolonged neurological and endocrine dysfunctions (15). Increasingly severe insomnia and anxiety are linked to more pronounced structural changes in the brain (16). Prior research has detected structural modifications in the prefrontal (17), temporal (18), and parietal lobes (19), as well as the hippocampus (20), anterior cingulate gyrus (21), and pineal gland areas (22) integral to sleep and emotion regulation. More recent studies have also noted abnormalities in the striatum (23) and locus coeruleus (24). FC analysis of these structurally abnormal regions allows for an examination of changes in functional activities, providing deeper insights into how structural abnormalities impact brain function and potentially elucidating the pathogenesis. Previous findings indicate abnormal FC in regions associated with sleep and emotional regulation, linked to both the ascending reticular activating system and the reward system (25). Additionally, some abnormalities correlate with the salience network and the negative emotional network (26), suggesting dysregulated sleep and emotional responses (27). The integration of VBM and FC analyses enhances our understanding of the pathogenesis of CID and GAD comorbidity and aids in the identification of further neural markers.

**Figure 1:**
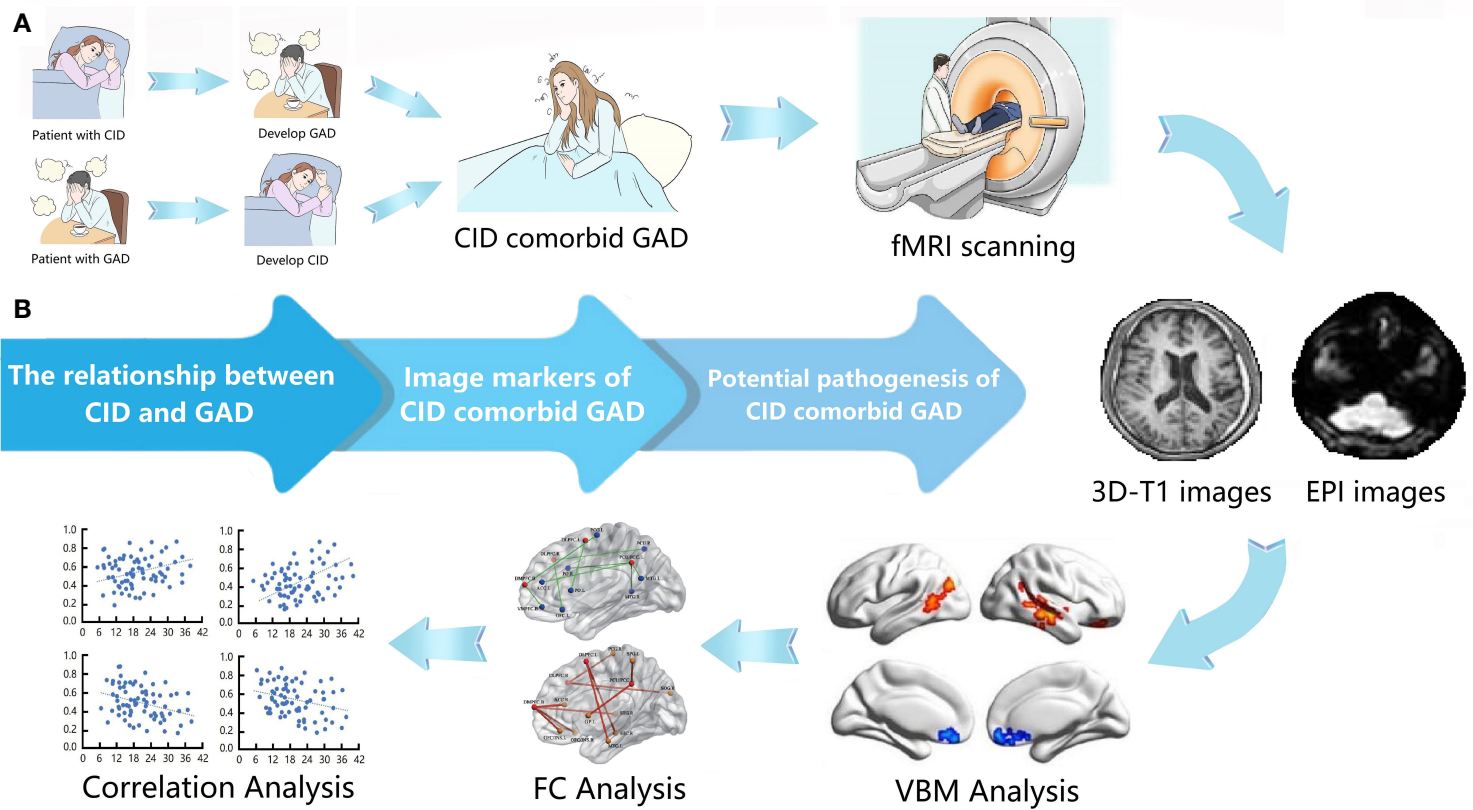
Overview of this study. **(A)** The process and steps of this study. **(B)** The purpose of this study. CID, chronic insomnia disorder; GAD, generalized anxiety disorder; VBM, voxel-based morphometry; FC, functional connectivity.

In this study, all participants diagnosed with both CID and GAD reflect these associations, consistent with previous research. Notably, nearly every patient with CID experiences sleep-related anxiety, a factor often overlooked in the literature. Our objective is to explore brain structure and functional changes attributable to CID and GAD, and to investigate the underlying causes of this comorbidity, with the hope of uncovering new directions for future research (**Figure 1**). Moreover, identifying neural markers of brain structure and function will facilitate a better understanding of its pathogenesis.

## 2 Materials and methods

### 2.1 Participants

In this study, a total of 57 patients were recruited from the Sleep and Psychiatry Center of the 960th Hospital. The International Classification of Sleep Disorders Third Edition (ICSD-3) and the Diagnostic and Statistical Manual of Mental Disorders Fifth Edition (DSM-5) were used as important diagnostic criteria. Two professional doctors confirmed that all patients suffer from CID comorbid GAD. Some key inclusion criteria include: (a) The Pittsburgh Sleep Quality Index (PSQI) scores >7, and the Insomnia Severity Index (ISI) scores >15; (b) The Self-rating Anxiety Scale (SAS) scores >50, and the Hamilton Anxiety Rating Scale (HAMA) scores >14; (c) Han national people aged 18–55 and right-handed. Some important exclusion criteria include: (a) Other psychiatric or sleep disorders; (b) Have taken medication, alcohol, cigarettes, or drugs within two weeks; (c) Severe brain structural abnormalities. (d) Contraindications of MRI scanning. In addition, 57 healthy people were recruited as healthy controls (HC). They meet some of the important criteria mentioned above.

This study complies with the Declaration of Helsinki and has been approved by the Research Ethics Committee of the 960th Hospital. All participants signed informed consent forms to indicate their agreement and knowledge of this study.

### 2.2 Diagnostic and differential criteria

According to the ICSD-3 diagnostic criteria, two experienced psychiatrists conduct the diagnosis of CID. Key diagnostic criteria include: (a) difficulty falling asleep and maintaining sleep; (b) Impaired capacity to function in daily life and at work; (c) persistent insomnia despite suitable sleep conditions; (d) insomnia occurring at least three times a week; (e) insomnia lasting for more than three months; (f) absence of other sleep disorders. Similarly, according to the DSM-5, two experienced psychiatrists diagnose GAD. Important diagnostic criteria include: (a) excessive anxiety about various events or activities; (b) difficulty controlling anxiety; (c) impaired capacity to function in daily life and at work; (d) anxiety persisting for more than six months; (e) manifestation of various physical and sleep symptoms; (f) absence of other mental or physical diseases. In this study, all patients with comorbid CID and GAD met the aforementioned criteria. as confirmed by two experienced psychiatrists.

### 2.3 Evaluation of clinical characteristics

Participants’ sleep quality and insomnia were assessed using the PSQI and ISI scales, while anxiety was evaluated through the SAS and HAMA scales. The Montreal Cognitive Assessment Scale (MoCA) confirmed that all participants possessed normal cognitive function. In these evaluations, the examiner-rated scales were assessed by two experienced psychiatrists, and the average scores were calculated. The self-rated scales were completed by the participants themselves under the guidance of psychiatrists. Finally, the results of these scales are reported as specific integer scores.

### 2.4 MRI data acquisition

MRI scanning was performed on a 3.0 T scanner (Discovery MR750, General Electric, Milwaukee, WI, United States) equipped with a standard eight-channel phased-array head coil. Participants lay supine with their heads secured using belts and pads to avoid head movements. During the fMRI scan, participants were required to close their eyes and relax without thinking or sleeping. The fMRI data were acquired using gradient echo-planar imaging sequence. Each scan consisted of 260 gradient echo-planar volumes with the parameters of: repetition time (TR)=2000 ms, echo time (TE)=30 ms, flip angle (FA)=90°, matrix=64×64, field of view (FOV)=24×24 cm^2^, voxel size=3×3×3 mm^3^, thickness/gap=4/0 mm, lasting 8 min 40 s. A three-dimensional brain volume imaging sequence was used for structural data acquisition with the parameters as follows: slices=132; TR=8.2 ms, TE=3.2 ms, slice thickness=1.0 mm, FOV=24×24 cm^2^, resolution=256×256, FA=12°, voxel size =1×1×1 mm^3^. In addition, Routine MRI sequences were performed to confirm no structural abnormalities in brain. Two professional doctors examined all MRI images to ensure quality compliance.

### 2.5 VBM data processing

Statistical Parametric Mapping 8 (SPM8, http://www.fil.ion.ucl.ac.uk/spm/) and its embedded toolbox on the MATLAB (The Math-Works Inc., Natick, MA, USA) were used to perform the structural data preprocessing and statistical analyses. The VBM processing steps are as follows: (1) Convert DICOM files to NIFITI images; (2) Screen for artifacts or anatomical abnormalities; (3) Manually re-oriented to the anterior; (4) Spatial registration of T1-weighted images to a reference brain template; (5) Segmentation of images into gray matter, white matter, and cerebrospinal fluid; (6) Transform the resulting images to standard Montreal Neurological Institute (MNI) space (http://www.mni.mcgill.ca/); (7) Perform the high-dimensional diffeomorphic anatomic registration through the exponentiated lie algebra (DARTEL) method for registration, normalization, and modulation; (8) The Jacobian determinants derived from spatial normalization that modulate the image intensity of each voxel were used to verify regional differences in the conservation of the total amount of gray matter; (9) Spatial Smoothing using a Gaussian kernel with a 4 mm full-width at half maximum (FWHM); (10) The gray matter volume (GMV) for each participant were obtained.

Two-sample t-tests was used to compare the GMV between groups. Age, gender, education years and total intracranial volume of participants were included as covariates. Gaussian Random Field (GRF) correction was used for multiple comparisons with corrected threshold of voxel p-value of < 0.005 and cluster p-value of < 0.05. Finally, GMV values of the brain areas with significant changes were extracted.

### 2.6 FC data processing

SPM8 and Resting-State fMRI Data Analysis 1.8 (Rest1.8, http://www.restfmri.net) toolbox on the MATLAB were used to perform the data processing and statistical analysis. The processing steps of FC are as follows: (1) Convert DICOM files into NIFITI images; (2) Discard the first 10 images; (3) Slice remaining images for time correction first, then realign to the first image and register with the average of these images; (4) Rigid-body head motion correction (2 mm displacement and 2 degrees rotation); (5) Segment individual T1 structural images (including white matter, gray matter, and cerebrospinal fluid); (6) Coregister each structural image with the functional image, and then reconstruct it into 3×3×3 mm^3^ resolution voxels; (7) Spatial normalization of the MNI; (8) Spatial smoothing using a Gaussian kernel with a 4 mm FWHM; (9) Remove the linear trend and perform 0.01–0.1 Hz band-pass filtering; (10) Nuisance covariates (head motion parameters, global mean signal, white matter signal and cerebrospinal fluid signal) were regressed from the data; (11) Define regions with significant group differences in GMV as regions of interest (ROIs) and seed regions. (12) Calculate the connectivity between the seed region and each voxel of the whole brain and, draw correlation maps; (13) Convert correlation maps into z-values maps using fisher’s r-to-z transformation.

Two-sample t-test was used to compare the FC differences between groups. Age, gender, and education years of participants were included as covariates. GRF correction was applied to perform multiple comparison correction. The significance threshold was set as voxel level of p-value < 0.005, and cluster level of p-value < 0.05. Finally, FC values of significantly changed brain areas were extracted.

### 2.7 Statistical analysis

Statistical Package For Social Sciences 20.0 (SPSS 20.0, Chicago, IL, USA) software was used for statistical analysis. Data were inspected for outliers, bias, and homogeneity of variance to ensure the appropriateness of parametric statistical tests. The independent two-sample t-test was performed to assess the differences in demographic and clinical characteristics (including age, education years, ISI scores, PSQI scores, HAMA scores, and SAS scores). The Chi-square test was performed to assess differences in gender. In addition, Pearson’s correlation analysis was performed to examine the relationship between clinical characteristics (including ISI scores, PSQI scores, HAMA scores, and SAS scores) and GMV or FC values. Age, gender, and education years of participants were included as covariates. Then, the relationship between different clinical characteristics also was examined. The statistical significance was set as *p*-value < 0.05.

## 3 Results

### 3.1 Demographic and clinical characteristics

**Figure 2:**
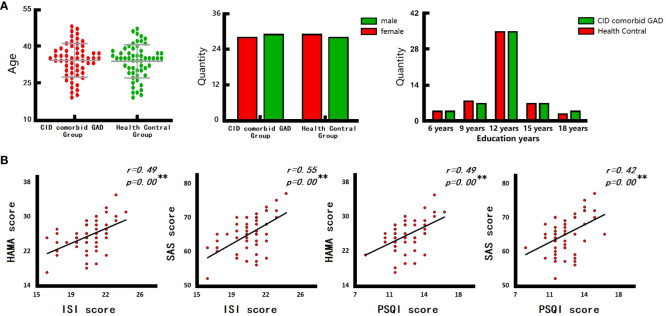
The results of clinical characteristics. **(A)** Age, gender, and education years of participants. **(B)** The relationship between CID and GAD. ISI, insomnia severity index scale; PSQI, pittsburgh sleep quality index; HAMA, hamilton anxiety scale; SAS, self-rating anxiety scale. ^*^p < 0.05, ^**^p < 0.01.

A total of 57 patients with CID comorbid GAD and 57 HCs were analyzed. No significant differences were found in gender, age, education years, MOCA score between groups. However, PSQI, ISI, HAMA, SAS scores of patients were significant higher than those of HCs (**Table 1**, **Figure 2A**).

**Table 1:** Demographic and clinical characteristics.

Characteristics	CID comorbid GAD (n = 57)	HC (n = 57)	t/χ²	*P* Value
Age (years old)	34.12 ± 6.86	33.77 ± 6.77	0.28^a^	0.78
Gender (M/F)	28/29	29/28	0.04^b^	0.85
Education (years)	11.84 ± 2.62	12.00 ± 2.72	0.32^a^	0.75
PSQI scores	12.04 ± 1.54	1.63 ± 0.56	48.12^a^	0.00^**^
ISI scores	20.19 ± 1.78	0.77 ± 0.76	75.91^a^	0.00^**^
HAMA scores	25.51 ± 3.50	0.51 ± 0.60	53.14^a^	0.00^**^
SAS scores	65.05 ± 5.35	0.42 ± 0.50	90.76^a^	0.00^**^
MoCA scores	29.89 ± 0.31	29.82 ± 0.38	1.08^a^	0.29

Values are reported as mean and standard deviation, except for gender. CID, chronic insomnia disorder; GAD, generalized anxiety disorder; HC, healthy control; M, male; F, female; PSQI, Pittsburgh Sleep Quality Index; ISI, Insomnia Severity Index Scale; HAMA, Hamilton Anxiety Scale; SAS, Self-rating Anxiety Scale; MoCA, Montreal Cognitive Assessment Scale. ^a^two-sample t-test, ^b^χ^2^-test, ^*^p < 0.05, ^**^p < 0.01.

### 3.2 Correlation analysis of clinical characteristics

There are close relationships between insomnia and anxiety in the group of CID comorbid GAD. Correlation analysis (**Figure 2B**) showed that, the ISI score of patients was significantly positively correlated with HAMA score (r=0.49, p=0.00) and SAS score (r=0.55, p=0.00). The PSQI score of patients was also significantly positively correlated with HAMA score (r=0.49, p=0.00) and SAS score (r=0.42, p=0.00).

### 3.3 VBM results

**Figure 3:**
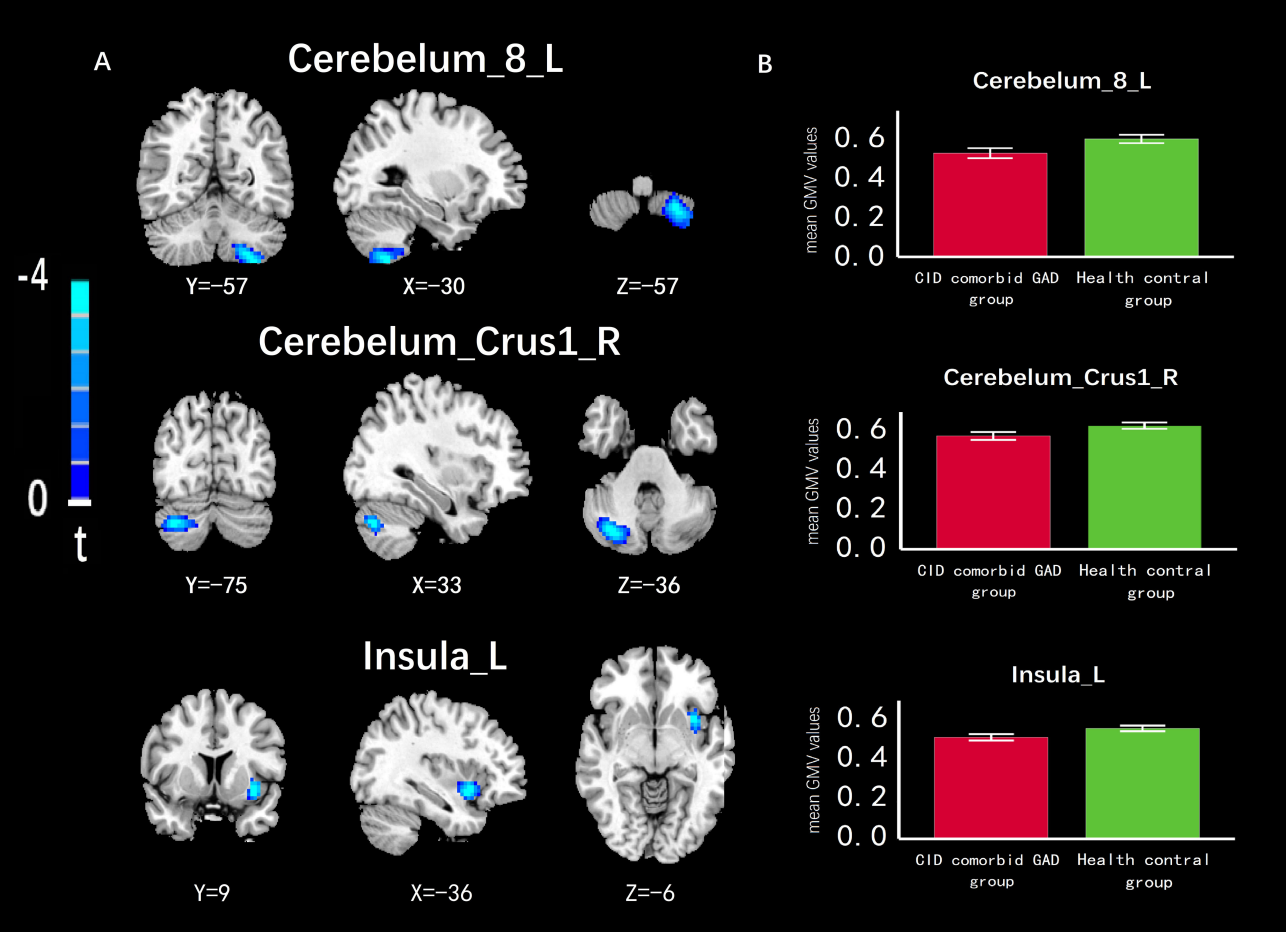
The difference of GMV in brain regions between groups. **(A)** The GMV of Cerebelum_8_L, Cerebelum_Crus1_R, Insula_L was significantly reduced in the group of CID comorbid GAD. **(B)** The difference of GMV values between groups. Errors bar showed standard error.

Compared with HC group, the GMV of left cerebellum (Cerebelum_8_L), right cerebellar peduncle (Cerebelum_Crus1_R), left insula (Insula_L) were significantly reduced in the group of CID comorbid GAD (**Figure 3**, **Table 2**).

**Table 2:** Results of VBM analyses.

Cluster regions	Volume	Cluster size (Voxels)	MNI coordinates			T value	*P* value
			X	Y	Z		
Cerebelum_8_L	reduce	269	-30	-57	-57	-4.02	0.01^**^
Cerebelum_Crus1_R	reduce	175	33	-75	-36	-3.94	0.01^**^
Insula_L	reduce	92	-36	9	-6	-3.94	0.00^**^

VBM, voxel-based morphometry; MNI, Montreal Neurological Institute; L, left; R, right. ^*^p < 0.05, ^**^p < 0.01.

### 3.4 FC results

**Figure 4:**
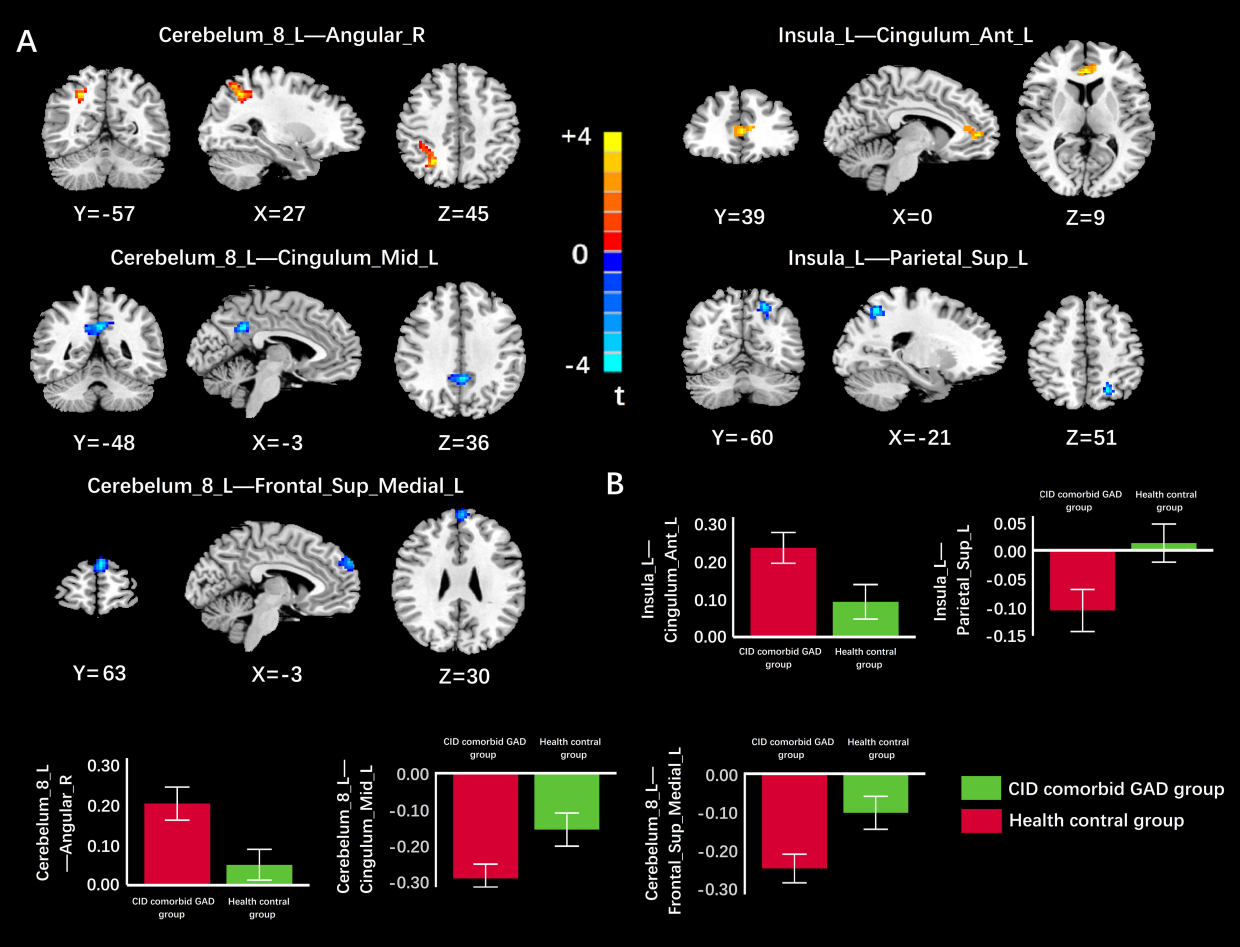
The difference of FC in brain regions between groups. **(A)** The FC of Cerebelum_8_L-Cingulum_Mid_L, Cerebelum_8_L-Frontal_Sup_Medial_L, Insula_L-Parietal_Sup_L was significantly reduced in the group of CID comorbid GAD. The FC of Cerebelum_8_L-Angular_R, Insula_L-Cingulum_Ant_L was significantly increased in the group of CID comorbid GAD. **(B)** The difference of FC z-values between groups. Errors bar showed standard error.

Based on VBM results, brain regions with GMV differences were defined as ROIs. Compared with HC group, functional connectivity between Cerebelum_8_L and middle left cingulate gyrus (Cingulum_Mid_L), between Cerebelum_8_L and left medial superior frontal gyrus (Frontal_Sup_Medial_L), between Insula_L and left superior postcentral gyrus (Parietal_Sup_L) were significantly reduced in the group of CID comorbid GAD. Functional connectivity between Cerebelum_8_L and right angular gyrus (Angular_R), between Insula_L and anterior left cingulate gyrus (Cingulum_Ant_L) were significantly increased (**Figure 4**, **Table 3**).

**Table 3:** Results of FC analyses.

ROIs	Brain regions	Cluster size (Voxels)	MNI coordinates			T value	*P* value
			X	Y	Z		
Cerebelum_8_L	Cingulum_Mid_L Frontal_Sup_Medial_L Angular_R	107 135 131	-3 -3 27	-48 63 -57	36 30 45	-4.55 -4.98 4.71	0.00^**^ 0.00^**^ 0.00^**^
Insula_L	Parietal_Sup_L Cingulum_Ant_L	97 137	-21 0	-60 39	51 9	-3.87 3.68	0.00^**^ 0.04^**^

ROIs, regions of interest; MNI, Montreal Neurological Institute; L, Left; R, Right. ^*^ *p* < 0.05, ^**^ *p* < 0.01.

### 3.5 Correlation analysis of GMV and FC values

**Figure 5:**
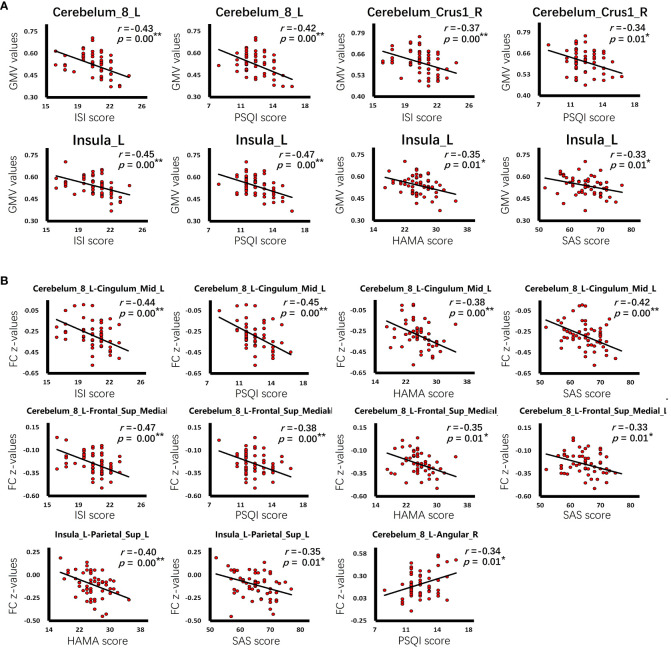
The correlation of clinical characteristics with GMV or FC. **(A)** The correlation of GMV values with insomnia or anxiety scale scores in the group of CID comorbid GAD. **(B)** The correlation of FC z-values with insomnia or anxiety scale scores in the group of CID comorbid GAD. ^*^ *p* < 0.05, ^**^ *p* < 0.01.

In the group of CID comorbid GAD, some brain regions were significantly associated with insomnia or anxiety (**Table 4** and **Figure 5**). The GMV of Cerebelum_8_L was significantly negatively correlated with the ISI scores (r=-0.43, p=0.00) and PSQI scores (r=-0.42, p=0.00). The GMV of Cerebelum_Crus1_R was significantly negatively correlated with the ISI scores (r=-0.37, p=0.00) and PSQI scores (r=-0.34, p=0.01). The GMV of Insula_L was significantly negatively correlated with the ISI scores (r=-0.45, p=0.00), PSQI scores (r=-0.47, p=0.00), HAMA scores (r=-0.35, p=0.01), SAS scores (r=-0.33, p=0.01). The FC of Cerebelum_8_L-Angular_R was significantly negatively correlated with the PSQI scores (r=-0.34, p=0.01). The FC of Cerebelum_8_L-Cingulum_Mid_L was significantly negatively correlated with the ISI scores (r=-0.44, p=0.00), PSQI scores (r=-0.45, p=0.00), HAMA scores (r=-0.38, p=0.00), SAS scores (r=-0.42, p=0.00). The FC of Cerebelum_8_L-Frontal_Sup_Medial_L was significantly negatively correlated with the ISI scores (r=-0.47, p=0.00), PSQI scores (r=-0.38, p=0.00), HAMA scores (r=-0.35, p=0.01), SAS scores (r=-0.33, p=0.01). The FC of Insula_L-Parietal_Sup_L was significantly negatively correlated with the HAMA scores (r=-0.40, p=0.00) and SAS scores (r=-0.35, p=0.01).

**Table 4:** Correlation between GMV/FC values and clinical characteristics.

VBM/FC values	clinical characteristics	r value	*p* value
Cerebelum_8_L (GMV)	ISI PSQI	-0.43 -0.42	0.00^**^ 0.00^**^
Cerebelum_Crus1_R (GMV)	ISI PSQI	-0.37 -0.34	0.00^**^ 0.01^*^
Insula_L (GMV)	ISI PSQI HAMA SAS	-0.45 -0.47 -0.35 -0.33	0.00^**^ 0.00^**^ 0.01^*^ 0.01^*^
Cerebelum_8_L-Angular_R (FC)	PSQI	-0.34	0.01^*^
Cerebelum_8_L-Cingulum_Mid_L (FC)	ISI PSQI HAMA SAS	-0.44 -0.45 -0.38 -0.42	0.00^**^ 0.00^**^ 0.00^**^ 0.00^**^
Cerebelum_8_L-Frontal_Sup_Medial_L (FC)	ISI PSQI HAMA SAS	-0.47 -0.38 -0.35 -0.33	0.00^**^ 0.00^**^ 0.01^*^ 0.01^*^
Insula_L-Parietal_Sup_L (FC)	HAMA SAS	-0.40 -0.35	0.00^**^ 0.01^*^

GMV, gray matter volume; FC, functional connectivity; ISI, insomnia severity index scale; PSQI, pittsburgh sleep quality index; HAMA, hamilton anxiety scale; SAS, self-rating anxiety scale. ^*^ *p* < 0.05, ^**^ *p* < 0.01.

## 4 Discussion

In this study, we have identified several key findings. Firstly, a positive correlation exists between the severity of insomnia and the severity of anxiety. Secondly, voxel-based morphologic analysis revealed a reduction in gray matter volume in the cerebellar and left insular regions of patients. Specifically, the reduction in cerebellar gray matter volume was negatively correlated with insomnia severity, while the reduction in the left insular gray matter volume was inversely correlated with both anxiety and insomnia severity. Thirdly, functional connectivity analysis indicated diminished connections between the left cerebellum and the left cingulate gyrus, between the left cerebellum and the left superior frontal gyrus, and between left insula and the left posterior central gyrus. Conversely, there were enhanced connections between the left cerebellum and the right angular gyrus, and between the left insula and the left anterior cingulate gyrus. These abnormal functional connections correlated with the severity scores of insomnia and anxiety, suggesting their relevance to the pathogenesis of insomnia comorbid with anxiety. Furthermore, these findings may also serve as potential neural markers and imaging features.

### 4.1 The relationship between insomnia and anxiety

In our study, there was a positive correlation between patients’ Pittsburgh Sleep Quality Index (PSQI) scores and Insomnia Severity Index (ISI) scores with their respective anxiety severity scores, as measured by the Hamilton Anxiety Rating Scale (HAMA) and the Self-Rating Anxiety Scale (SAS). This suggests that insomnia and anxiety are intricately linked, often appearing as comorbid conditions. Supporting this, a large-scale study has established a causal relationship between the two disorders, where insomnia frequently acts as a precursor to anxiety (28). Moreover, longitudinal research has demonstrated that chronic insomnia can predict subsequent anxiety development (29), and that treating insomnia effectively improves sleep quality and reduces anxiety symptoms (30). Typically, individuals with anxiety experience reduced sleep duration and disrupted sleep continuity (14). These findings underscore the importance of closely monitoring insomnia symptoms in patients with anxiety (31). Both disorders are highly prevalent and were found to nearly triple in incidence during the COVID-19 pandemic (32), with many individuals experiencing both simultaneously (33). Therefore, it is crucial to explore their pathogenesis further, given the extensive impact on the population.

Previous researchers have posited various causes for the comorbidity of insomnia and anxiety. They initially hypothesized that insomnia stems from a state of hyperarousal in the brain. It has been suggested that abnormalities in the brainstem’s ascending reticular activation system contribute to these conditions, affecting not only sleep but also emotional regulation. Specifically, these abnormalities may lead to both insomnia and anxiety (34). Further research identified issues within the emotional regulation and arousal circuits of patients with insomnia, highlighting a potentially significant role for the locus coeruleus. This suggests that insomnia may trigger hyperactivity in the locus coeruleus at night, impeding anxiety relief and exacerbating the condition, thus perpetuating the cycle of comorbid insomnia and anxiety (35). The regulation of emotions and sleep is also influenced by hormonal activity, with norepinephrine playing a key role in maintaining states of arousal and anxiety. Abnormalities in the locus coeruleus-norepinephrine system have been closely linked to both insomnia and anxiety (36). Additionally, special neuronal abnormalities are implicated in this comorbidity. For instance, glutaminergic neurons, which regulate both emotions and sleep, have been found to malfunction; abnormalities in the glutaminergic neurons-cingulate cortex circuit are associated with anxiety, while issues in the glutaminergic neuron-paraventricular thalamic nucleus circuit are linked to insomnia (37). In summary, the comorbidity of insomnia and anxiety may arise from shared pathological mechanisms, a finding consistent with our research. From the perspective of GMV and FC, we tried to discover the mechanism of insomnia comorbidity anxiety. In addition, we also hope to discover neural markers and Image features.

### 4.2 The abnormal change in brain structure

In our study, the reduction in cerebellar gray matter volume may serve as neural marker and Image feature, showing a negative correlation with the severity of insomnia. The cerebellum’s role extends beyond maintaining body balance, regulating muscle tone, and coordinating voluntary movements. Experimental evidence, such as the increased drowsiness observed in cerebellectomized cats (38) and changes in neuronal activity in mice correlating with sleep-wake transitions, supports its involvement in sleep regulation (39). Further research has demonstrated that the cerebellum has extensive connections with other brain regions during sleep and may influence the activity of areas associated with sleep regulation. For instance, during certain sleep phases, the electrophysiological activity of the cerebellum mirrors that of the hippocampus, suggesting a collaborative role in sleep management (40). Moreover, clinical observations indicate that patients with cerebellar damage often exhibit sleep disturbances (41), highlighting a significant relationship between the cerebellum and sleep. Thus, the cerebellum may function as a relay station in sleep regulation, receiving signals from and impacting various brain regions (42). Our findings suggest that a decrease in cerebellar gray matter volume is morphologically associated with insomnia.

The abnormal volume of the insula may also serve as a neural marker. The observed decrease in insular gray matter volume appears to be a consequence of comorbid insomnia and anxiety. We found that this decrease in the left insula was negatively correlated with the Insomnia Severity Index (ISI) scores (r=-0.45) and Hamilton Anxiety Rating Scale (HAMA) scores (r=-0.35). The insula is crucial for the regulation of human emotions, functioning in conjunction with brain regions such as the amygdala, thalamus, and prefrontal lobe. Moreover, the insula influences emotional regulation through various mechanisms, including the modulation of hormones and neurotransmitters and alterations in the functional states of certain brain regions (43). Abnormalities in the insula are prevalent in many mental disorders (44), with anxiety exerting a significant and persistent impact on insular gray matter volume. Notably, the volume of insular gray matter tends to decreases as the severity of anxiety increases (45). However, anxiety is not the sole cause of insular atrophy. Research indicates that changes in brain structure in anxious patients are also negatively correlated with the severity of their insomnia (46), suggesting that insomnia contributes to insular abnormalities as well. Indeed, some studies demonstrated that the insula mediates the onset of both anxiety and insomnia. Under significant stress, the functional state of the insula typically becomes abnormal, which may lead to both anxiety and insomnia (47). Additionally, experimental activation of insular activity has been shown to induce anxiety and sleep disorders (48). We propose that the loss of gray matter volume in the insula may be associated with the comorbidity of insomnia and anxiety.

### 4.3 The abnormal change in brain function

We observed abnormalities in functional connectivity that may reflect changes in the functional brain activity of patients and could also serve as image features. Specifically, the connections between the left cerebellum and the left cingulate gyrus, as well as between the left cerebellum and the left superior frontal gyrus, were diminished. These reductions were negatively correlated with the severity of both insomnia and anxiety. Conversely, connectivity between the left insula and the left cingulate gyrus was enhanced. Such abnormal functional connections likely represent disturbances in emotional and sleep regulation among patients The cingulate gyrus and the superior frontal gyrus are critical neural nodes in the mechanisms of emotional regulation and are also implicated in sleep processes. Studies have shown that prolonged poor sleep quality can reduce gray matter volume in the cingulate gyrus (49). Additionally, the role of the prefrontal lobe in sleep arousal has been emphasized in numerous studies (50). Insomnia not only affects the superior frontal gyrus and cingulate gyrus by causing abnormalities but also leads to decreased myelin levels in the cortex (51) and altered neurotransmitter levels. Prolonged insomnia may also disrupt the human body’s default network, negative emotional network, and reward network (52), all of which are closely associated with the superior frontal gyrus and cingulate gyrus. Consequently, these abnormalities could precipitate anxiety. Similar findings have been reported in previous research, which noted that disturbances in the functional connectivity of the superior frontal and cingulate gyrus are linked to both insomnia and anxiety (53, 54). We propose that these abnormal functional connections may be significant neural markers.

In addition, we posit those abnormal functional connections in certain brain regions may elucidate the somatization symptoms associated with insomnia and anxiety. Specifically, the reduced connectivity between the left insula and the left upper posterior central gyrus was negatively correlated with anxiety severity. Conversely, the increased connections between the left cerebellum and the right angular gyrus were negatively correlated with insomnia severity, potentially linked to complications observed in patients with either disorder. Commonly reported symptoms among these patients include fatigue, tension, muscle soreness, memory loss, and concentration impairment (55). The posterior central gyrus, responsible for somatosensory functions, when impaired, is typically associated with symptoms such as numbness, heaviness, and sensory weakness. Functional abnormalities in this region among anxiety sufferers are well-documented and are believed to contribute to their physical symptoms. Moreover, the dysfunction of the posterior central gyrus may facilitate the progression from anxiety to depression (56), underscoring its role beyond mere somatosensory functions to include emotional regulation. Indeed, previous cases of posterior central gyrus injury have exhibited emotional regulation impairments, likely due to its neural connections with the amygdala and insula (57), although the exact mechanisms remain to be clarified. The angular gyrus, known as the visual language center, is integral for processing written and spoken information. Abnormalities in this region might manifest as diminished comprehension (58), which can be linked to the memory and attention deficits associated with chronic insomnia.

### 4.4 Limitations of this study

Our study is subject to several limitations. Firstly, it employs a cross-sectional design with participants undergoing only a single MRI scan, which does not allow for tracking the progression of the disease over time. Secondly, integrating analyses of neurotransmitter levels, neural cell integrity, and genetic data with imaging features would be highly beneficial; we aim to address these aspects in future studies. Thirdly, increasing the sample size would enhance the reliability of our findings and enable the design of more experimental groups. A notable limitation is the absence of discrete groups for patients with only insomnia or only anxiety, which restricts our ability to thoroughly explore the mechanisms specific to each condition. Moving forward, we plan to recruit a broader cohort of patients to facilitate more detailed investigations into these disorders. Further research is already being planned by our team.

## 5 Conclusions

In this study, we have identified three main conclusions, aiming to inspire future research. Firstly, we posit that there is a close relationship between insomnia and anxiety, with the severity of insomnia in patients being positively correlated with the severity of anxiety. Secondly, the GMV of cerebellum and insula was found to be reduced in patients. The reduction in cerebellar GMV could be attributable to long-term insomnia and might serve as an imaging marker of CID. Similarly, the reduced GMV of the insula, potentially due to anxiety and insomnia, could play a role in the pathophysiology of CID comorbid with GAD. Thirdly, functional abnormalities were observed in the cingulate gyrus, prefrontal lobe, postcentral gyrus, and angular gyrus of patients. These abnormalities in the cingulate gyrus and prefrontal lobe could be implicated in the pathogenesis of CID comorbid with GAD and are also considered potential imaging markers. Moreover, abnormalities in the Postcentral gyrus and angular gyrus may elucidate common clinical symptoms in patients with CID comorbid GAD.

## Data availability statement

The raw data supporting the conclusions of this article will be made available by the authors, without undue reservation.

## Ethics statement

The studies involving humans were approved by Research Ethics Committee of the 960th Hospital of the People’s Liberation Army Joint Logistic Support Force. The studies were conducted in accordance with the local legislation and institutional requirements. The participants provided their written informed consent to participate in this study. Written informed consent was obtained from the individual(s) for the publication of any potentially identifiable images or data included in this article.

## Author contributions

MX: Writing – original draft, Data curation. BL: Writing – original draft, Formal analysis. SW: Writing – original draft, Investigation. CC: Writing – original draft, Investigation. ZL: Writing – original draft, Investigation. YJ: Writing – original draft, Investigation. KL: Writing – review & editing, Conceptualization. YN: Writing – original draft, Conceptualization.
